# Effects of Luteolin in an In Vitro Model of Porcine Intestinal Infections

**DOI:** 10.3390/ani14131952

**Published:** 2024-07-02

**Authors:** Dóra Kovács, Nikolett Palkovicsné Pézsa, Alma Virág Móritz, Ákos Jerzsele, Orsolya Farkas

**Affiliations:** 1 Department of Pharmacology and Toxicology, University of Veterinary Medicine, 1078 Budapest, Hungary; palkovicsne.pezsa.nikolett@univet.hu (N.P.P.); moritz.alma.virag@univet.hu (A.V.M.); jerzsele.akos@univet.hu (Á.J.); farkas.orsolya@univet.hu (O.F.); 2 National Laboratory of Infectious Animal Diseases, Antimicrobial Resistance, Veterinary Public Health and Food Chain Safety, University of Veterinary Medicine, 1078 Budapest, Hungary

**Keywords:** luteolin, flavonoids, pigs, porcine, intestines, IPEC-J2, bacterial infections, *Escherichia coli*, *Salmonella enterica*, AMR

## Abstract

**Simple Summary:**

Diarrhea, caused by bacteria such as *Escherichia coli* and *Salmonella enterica*, is a common health problem in pigs. Antibiotics are generally used for the treatment of these infections; however, their efficacy can be compromised via bacterial resistance. Thus, there is a growing need for alternative substances and methods for preventing and treating these diseases. The aim of this research was to test the beneficial effects of a natural substance, luteolin, in *E. coli* and *S*. *enterica* subsp. *enterica* serovar Typhimurium infections. For this purpose, we used a swine intestinal cell culture model that was infected with bacteria with or without the addition of luteolin. In our experiments, luteolin showed various effects that could be beneficial for the treatment of *E. coli* and *S*. Typhimurium infections in pigs. Luteolin alleviated oxidative stress and inflammation, and protected the integrity of the cell layer. Based on these findings, luteolin could be used in *E. coli* and *S.* Typhimurium diarrhea in pigs to counteract the deleterious effects of bacteria in the intestines; however, further studies are needed to confirm its efficacy. The use of luteolin in the future could ultimately lead to a reduced need for antibiotics in pig production.

**Abstract:**

Intestinal infections caused by *Escherichia coli* and *Salmonella enterica* pose a huge economic burden on the swine industry that is exacerbated by the development of antimicrobial resistance in these pathogens, thus raising the need for alternative prevention and treatment methods. Our aim was to test the beneficial effects of the flavonoid luteolin in an in vitro model of porcine intestinal infections. We infected the porcine intestinal epithelial cell line IPEC-J2 with *E. coli* and *S*. *enterica* subsp. *enterica* serovar Typhimurium (10^6^ CFU/mL) with or without previous, concurrent, or subsequent treatment with luteolin (25 or 50 µg/mL), and measured the changes in the reactive oxygen species and interleukin-6 and -8 levels of cells. We also tested the ability of luteolin to inhibit the adhesion of bacteria to the cell layer, and to counteract the barrier integrity damage caused by the pathogens. Luteolin was able to alleviate oxidative stress, inflammation, and barrier integrity damage, but it could not inhibit the adhesion of bacteria to IPEC-J2 cells. Luteolin is a promising candidate to be used in intestinal infections of pigs, however, further studies are needed to confirm its efficacy. The use of luteolin in the future could ultimately lead to a reduced need for antibiotics in pig production.

## 1. Introduction

Diarrhea is one of the most common diseases of pigs worldwide [1], with *Escherichia coli* and *Salmonella enterica* being among the main causative agents [2,3]. Enterotoxigenic *E. coli* (ETEC) strains commonly cause diarrhea in neonatal and weaned piglets [1,4]. The pathogenesis of ETEC infections in the intestines is initiated by the attachment of bacteria to the intestinal epithelium [1,2,5,6], which is followed by colonization and the secretion of enterotoxins that induce inflammation and damage the intestinal barrier integrity [2,6]. Similarly, salmonellosis occurs most commonly in recently weaned piglets but in growing-finishing pigs as well [3]. Infections with *S. enterica* also start with the attachment of bacteria to the intestinal epithelial cell surface [7], which leads to inflammation and the production of reactive oxygen species (ROS), as well as the disruption of tight junctions in the intestines [3,7]. The impairment of the intestinal barrier function in *E. coli* and *Salmonella* spp. infections may lead to the translocation of bacteria to the systemic circulation [8], thus exacerbating the disease. Infections caused by *E. coli* and *S*. *enterica* are associated with significant economic losses to the swine industry due to the reduced growth rate and mortality of animals [1,3,4,5,7,9]. In addition, both bacteria can cause foodborne zoonoses, thus posing a concern for public health [3,7]. As pork is among the most popular meats worldwide [4], it is of global interest to effectively control these infections.

Antimicrobials are widely used for the prevention and treatment of intestinal diseases in pigs, including infections caused by *E. coli* and *Salmonella* spp. However, the spread of resistant bacterial strains makes these treatments increasingly difficult [3,4]. In addition, resistant bacteria evolved in food-producing animals can spread to humans through the food chain, direct contact, and indirectly via the environment [10,11,12], thus posing a threat to human health as well. As AMR is a rising concern for animal, human, and environmental health, various national and international regulations and guidelines are in place to limit the use of antimicrobials in livestock. Consequently, there is an increasing need for developing substances or measures alternative to antimicrobials for the prevention and treatment of intestinal bacterial infections [2,3,4,5,6,9,13,14]. Potential antimicrobial alternatives include—among others—vaccines, bacteriophages, antimicrobial peptides, phytochemicals, organic acids, and pre-, pro-, and synbiotics [3,4,5,6,14]. Phytochemicals, such as flavonoids, are secondary plant metabolites with various beneficial properties, including antioxidant, anti-inflammatory, antiviral, and antibacterial effects. Phytochemicals can be extracted from plants or synthesized directly [2].

Although research data are available on the efficiency of potential antimicrobial alternatives, there is still a need to better understand their mechanism of action in bacterial infections, and the limitations of their use. In vivo animal experiments are great tools to carry out research on the effect of antimicrobial alternatives, however, for technical, financial, and ethical reasons, in vitro models should be considered. The use of in vitro systems provides a time- and cost-effective solution for experiments with good reproducibility [4]. For instance, cell cultures are valuable tools for studying the effects of phytochemicals on the gastrointestinal tract in vitro [15]. Among the available cell lines, IPEC-J2 is widely used for modeling intestinal bacterial infections and for testing the effect of various substances on intestinal inflammation, oxidative stress, and barrier function [16]. IPEC-J2 is a non-transformed, non-tumorigenic intestinal epithelial cell line originating from the jejunum of a neonatal, unsuckled piglet that shows high morphological and functional similarity with porcine epithelial cells in vivo [16,17].

Luteolin (3′,4′,5,7-tetrahydroxyflavone, LUT) is a flavonoid belonging to the group of flavones that is present in various plants, vegetables, herbs, and fruits, and that shows several effects with potential health benefits, such as anti-inflammatory, antioxidant, antimicrobial, anti-allergic, anticancer, cardioprotective, antidiabetic, and neuroprotective activities [18,19,20,21,22]. LUT may occur in glycoside and aglycone forms, among which the glycoside form is more abundant in natural sources, while the aglycone possesses more potent antioxidant and anti-inflammatory capacities [19]. Most of the studies with LUT focus on the beneficial effects of the flavonoid on human health, while less information is available about its activity in animals. It should also be noted that LUT can act as a pro-oxidant under certain circumstances [23,24], and its activity may depend on the cell type and the concentration being tested [25]. Therefore, it is important to test the effects of LUT in various experimental settings and concentrations.

The aim of this research was to evaluate the potential of LUT to counteract the deleterious effects of *E. coli* and *S. enterica* in porcine intestinal cells, including oxidative stress, inflammation, barrier integrity damage, and the adhesion of bacteria to the cells. These effects of LUT could be beneficial for the treatment of intestinal bacterial infections in pigs and could ultimately reduce the need for antibiotics in pig production.

## 2. Materials and Methods

### 2.1. Culture Conditions

To test the potential beneficial effects of LUT in intestinal infections of pigs, we used an in vitro model of porcine intestinal infections, i.e., porcine intestinal epithelial cells challenged with *E. coli* and *S. enterica* subsp. *enterica* serovar Typhimurium. For all experiments, we used the IPEC-J2 porcine intestinal epithelial cell line (provided by Dr. Jody Gookin from the North Carolina State University), and *E. coli* and *S*. Typhimurium field isolates from pigs with gastrointestinal infections.

Prior to the experiments, we cultured IPEC-J2 cells until the 50th passage in the mixture (1:1) of Dulbecco’s Modified Eagle’s Medium and Ham’s F-12 Nutrient (DMEM/F12, from Sigma-Aldrich [Darmstadt, Germany]) supplemented with 5% fetal bovine serum, 5 ng/mL of epidermal growth factor, 5 µg/mL of insulin, 100 IU/mL of penicillin-streptomycin, 5 µg/mL of transferrin, and 5 ng/mL of selenium (full DMEM/F12). Before each test, the cells were cultured until forming a differentiated, confluent monolayer in the cell culture plates. During the experiments, the culture medium was used without supplementation (plain DMEM/F12) for preparing the working solutions. When the treatments were followed by an incubation period before a specific measurement, the cells were kept in DMEM/F12 supplemented with penicillin–streptomycin to avoid bacterial overgrowth. In all cases prior to and during the experiments, the IPEC-J2 cells were kept at 37 °C, with 5% carbon dioxide in their environment.

The *E. coli* and *S*. Typhimurium strains were kept frozen (at −80 °C in Microbank tubes) before the experiments. In 18–24 h prior to the experiments, they were propagated in plain DMEM/12 in a similar environment as the IPEC-J2 cells (37 °C, 5% carbon dioxide).

### 2.2. Treatment Scheme

The same treatment scheme was used in all experiments. Positive control cells were challenged with either *E. coli* or *S*. Typhimurium at the concentration of 10^6^ colony-forming units (CFU)/mL for both bacteria to provoke oxidative stress, inflammation, and barrier integrity damage in the IPEC-J2 cells. To determine the potential antioxidant, anti-inflammatory, barrier-protective, and anti-adhesive effects of LUT (Sigma-Aldrich) against these bacteria, different groups of IPEC-J2 cells were treated with LUT in two different concentrations (25 and 50 µg/mL) either before, concurrently, or after infection with bacteria. In “Group A”, the LUT treatment preceded the addition of bacteria; cells in “Group B” were treated simultaneously with LUT and bacteria; and in “Group C”, cells were first infected with bacteria and then treated with LUT. Cells kept in plain DMEM/F12 served as a negative control. The treatment groups are summarized in Table 1. All treatments lasted for one hour and were followed by rinsing with phosphate-buffered saline (PBS). The tested concentrations of LUT and the bacterial suspensions were chosen based on previous studies. When applied for one hour on IPEC-J2 cells, these concentrations of LUT and the bacteria did not have a negative effect on cell viability in previous experiments [26,27].

### 2.3. Measurements

The potential antioxidant effect of LUT was tested with the dichloro-dihydro-fluorescein diacetate (DCFH-DA) assay (Sigma-Aldrich). The above-described treatments were performed on IPEC-J2 cells cultured on 6-well polystyrene cell culture plates (Corning Inc. [Corning, NY, USA]), followed by one day of incubation before the measurement. DCFH-DA reagent was applied on the cells for one hour, followed by rinsing, scraping, centrifugation, and fluorescence measurements (SpectraMax iD3, Molecular Devices [San José, CA, USA]), as described previously [26,27]. DCFH-DA is oxidized by intracellular ROS (IC ROS) to the fluorescent dichlorofluorescein (DCF) [28]; therefore, measuring higher fluorescence values suggests the presence of more ROS in the system.

To test the anti-inflammatory properties of LUT, the interleukin-6 and interleukin-8 (IL-6 and IL-8) levels of the cells were measured with enzyme-linked immunosorbent assay (ELISA). For these experiments, cells were propagated on 6-well polystyrene cell culture plates, followed by treatments according to Table 1, and sampling from the cell supernatants six hours later [29,30,31]. The samples were tested with porcine-specific ELISA kits (Sigma-Aldrich) as per the user instructions, and the absorbance measurement was performed with SpectraMax iD3.

To determine the paracellular permeability of the cell layers and, therefore, to estimate the impact of bacteria and LUT on barrier integrity, the fluorescein isothiocyanate–dextran 4 kDa (FD4) tracer dye (Sigma-Aldrich) was used. For these tests, cells were cultured on 12-well polyester membrane inserts (pore size: 0.4 µm, Corning Inc.), and their transepithelial electrical resistance (TEER) was measured regularly before the experiments. After the treatments were performed according to Table 1, 0.25 mg/mL of FD4 was applied on the cells, and samples were taken from the basolateral compartment of the wells three hours and one day after the end of the treatments. The fluorescence of these samples was measured with SpectraMax iD3 to detect the amount of dye that passed through the cell layer, indicating the level of barrier integrity.

To test whether LUT can inhibit the adhesion of bacteria to IPEC-J2 cells, the CFU count of bacteria attached to the cells was determined using selective agar plates. For these experiments, cells grown on 24-well polystyrene cell culture plates (Corning Inc.) were used. At the end of the treatments (as per Table 1), the supernatants of the cells were removed to eliminate the bacteria not attached to the cells, and 1% Triton X (Sigma-Aldrich) was applied to lyse the cells and, therefore, release those bacteria that could adhere to the cells. The lysis lasted for half an hour, and the plates were kept in a shaker to enhance the process. Subsequently, a serial dilution was created using the homogenized suspensions from each well and was inoculated onto selective agar plates (ChromoBio Coliform for *E. coli* and ChromoBio Salmonella Plus for *S*. Typhimurium, Biolab Zrt. [Budapest, Hungary]). The plates were then left to incubate overnight, and the following day, the CFUs were enumerated.

### 2.4. Statistics

Statistical analysis of the data was performed with R 3.3.2 (2016) software (R Foundation for Statistical Computing, Vienna, Austria) using one-way ANOVA and Tukey’s post hoc test to compare the mean values of different treatment groups. The results were considered significant in the case of a *p* < 0.05.

## 3. Results

### 3.1. Antioxidant Activity

**Figure 1:**
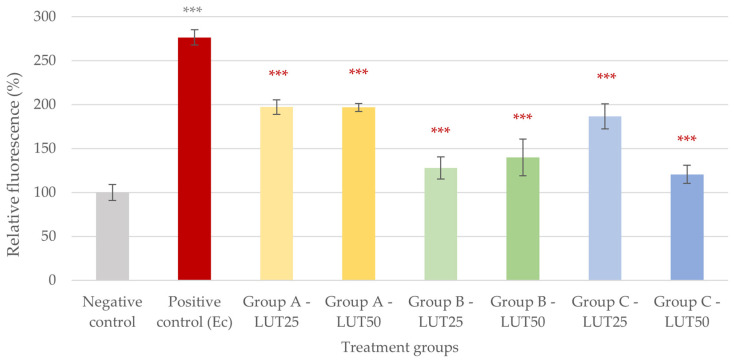
Intracellular reactive oxygen species level of IPEC-J2 cells after one-hour treatment with *Escherichia coli* and luteolin (LUT). Negative control: untreated cells (plain medium only); positive control: cells challenged with 10^6^ CFU/mL *E. coli*. Group A—LUT25 or LUT50: cells treated with 25 or 50 μg/mL LUT before challenge with 10^6^ CFU/mL *E. coli*; Group B—LUT25 or LUT50: cells treated with 25 or 50 μg/mL LUT concurrently with challenge with 10^6^ CFU/mL *E. coli*; Group C—LUT25 or LUT50: cells treated with 25 or 50 μg/mL LUT after challenge with 10^6^ CFU/mL *E. coli*. Data are shown as means with standard deviation and expressed as relative fluorescence, considering the mean value of control as 100%. N = 6/group. Significant difference: *** *p* < 0.001, asterisk in gray: compared to the negative (untreated) control; in red: compared to the positive control (*E. coli* challenge).

**Figure 2:**
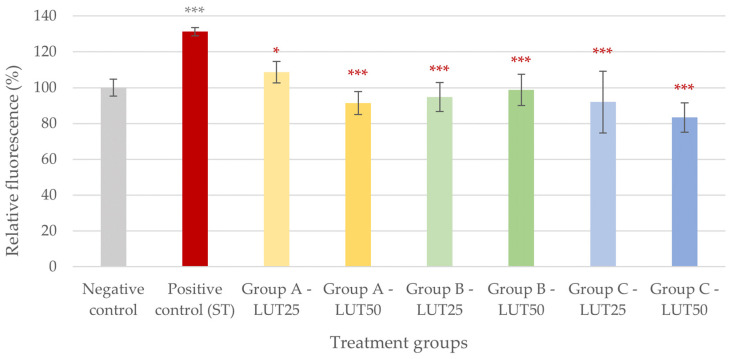
Intracellular reactive oxygen species level of IPEC-J2 cells after one-hour treatment with *Salmonella* Typhimurium and luteolin (LUT). Negative control: untreated cells (plain medium only); positive control: cells challenged with 10^6^ CFU/mL *S.* Typhimurium. Group A—LUT25 or LUT50: cells treated with 25 or 50 μg/mL LUT before challenge with 10^6^ CFU/mL *S.* Typhimurium; Group B—LUT25 or LUT50: cells treated with 25 or 50 μg/mL LUT concurrently with challenge with 10^6^ CFU/mL *S.* Typhimurium; Group C—LUT25 or LUT50: cells treated with 25 or 50 μg/mL LUT after challenge with 10^6^ CFU/mL *S.* Typhimurium. Data are shown as means with standard deviation and expressed as relative fluorescence, considering the mean value of control as 100%. N = 6/group. Significant difference: * *p* < 0.05, *** *p* < 0.001, asterisk in gray: compared to the negative (untreated) control; in red: compared to the positive control (*S.* Typhimurium challenge).

The IC ROS level of the IPEC-J2 cells significantly increased due to challenges with both *E. coli* and *S*. Typhimurium (Figure 1 and Figure 2). The addition of LUT in all groups and concentrations resulted in a decrease in these ROS levels, suggesting a potent antioxidant effect of LUT against infections caused by both bacterial species. In the case of *E. coli*, the activity of LUT was not impacted by its concentration in Groups A and B, while the higher concentration of LUT had a more pronounced activity than the lower concentration when applied after the challenge with bacteria (Group C, *p* < 0.001). When comparing the treatment types, concurrent LUT treatment (Group B) seemed to be the most effective in the case of 25 μg/mL of LUT (*p* < 0.001). For 50 μg/mL of LUT, treatments concurrently with (Group B) and after the bacterial infection (Group C) showed more potent activity than treatment before the bacterial challenge (Group A, *p* < 0.001) (Figure 1).

In the case of oxidative stress caused by *S*. Typhimurium, all LUT treatments had similar efficacies in decreasing the IC ROS levels. There was no difference in the antioxidant activity of LUT regardless of the treatment type (Group A, B, or C) and the applied concentration (Figure 2).

### 3.2. Anti-Inflammatory Activity

**Figure 3:**
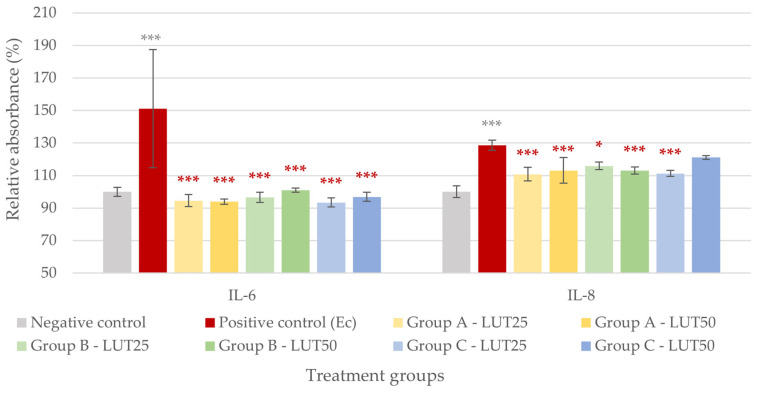
Interleukin-6 and interleukin-8 levels of IPEC-J2 cells after one-hour treatment with *Escherichia coli* and luteolin (LUT). Negative control: untreated cells (plain medium only); positive control: cells challenged with 10^6^ CFU/mL *E. coli*. Group A—LUT25 or LUT50: cells treated with 25 or 50 μg/mL LUT before challenge with 10^6^ CFU/mL *E. coli*; Group B—LUT25 or LUT50: cells treated with 25 or 50 μg/mL LUT concurrently with challenge with 10^6^ CFU/mL *E. coli*; Group C—LUT25 or LUT50: cells treated with 25 or 50 μg/mL LUT after challenge with 10^6^ CFU/mL *E. coli*. Data are shown as means with standard deviation and expressed as relative absorbance, considering the mean value of control as 100%. N = 6/group. Significant difference: * *p* < 0.5, *** *p* < 0.001, asterisk in gray: compared to the negative (untreated) control; in red: compared to the positive control (*E. coli* challenge).

**Figure 4:**
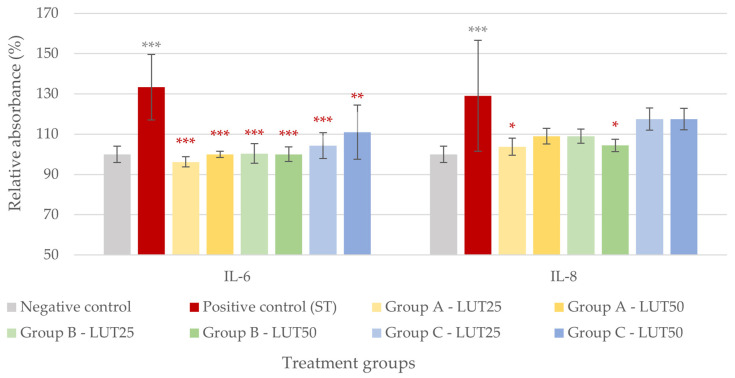
Interleukin-6 and interleukin-8 levels of IPEC-J2 cells after one-hour treatment with *Salmonella* Typhimurium and luteolin (LUT). Negative control: untreated cells (plain medium only); positive control: cells challenged with 10^6^ CFU/mL *S.* Typhimurium. Group A—LUT25 or LUT50: cells treated with 25 or 50 μg/mL LUT before challenge with 10^6^ CFU/mL *S.* Typhimurium; Group B—LUT25 or LUT50: cells treated with 25 or 50 μg/mL LUT concurrently with challenge with 10^6^ CFU/mL *S.* Typhimurium; Group C—LUT25 or LUT50: cells treated with 25 or 50 μg/mL LUT after challenge with 10^6^ CFU/mL *S.* Typhimurium. Data are shown as means with standard deviation and expressed as relative absorbance, considering the mean value of control as 100%. N = 6/group. Significant difference: * *p* < 0.5, ** *p* < 0.01, *** *p* < 0.001, asterisk in gray: compared to the negative (untreated) control; in red: compared to the positive control (*S.* Typhimurium challenge).

Significantly increased IL-6 and IL-8 levels were measured in the IPEC-J2 cells after the challenge with *E. coli* and *S*. Typhimurium (Figure 3 and Figure 4). When LUT was applied in combination with *E. coli*, it showed a significant anti-inflammatory effect. All LUT treatments (Groups A, B, and C, with 25 and 50 μg/mL LUT each) could decrease the IL-6 levels elevated by the bacteria, and the treatment types had similar efficacy. All LUT treatments similarly reduced the IL-8 production of cells, except for the higher concentration when used after the challenge with bacteria (Group C). The results are shown in Figure 3.

Similarly to the observations with *E. coli*, LUT showed an anti-inflammatory effect in the case of the *S*. Typhimurium challenge. All LUT treatments could significantly decrease the IL-6 levels elevated by the challenges with *S*. Typhimurium, and the various treatment types did not differ in their efficacy. However, only two LUT treatments could significantly reduce the IL-8 levels: 25 μg/mL of LUT applied before bacteria (Group A) and 50 μg/mL of LUT added concurrently with bacteria (Group B) (Figure 4).

### 3.3. Barrier Protection

**Figure 5:**
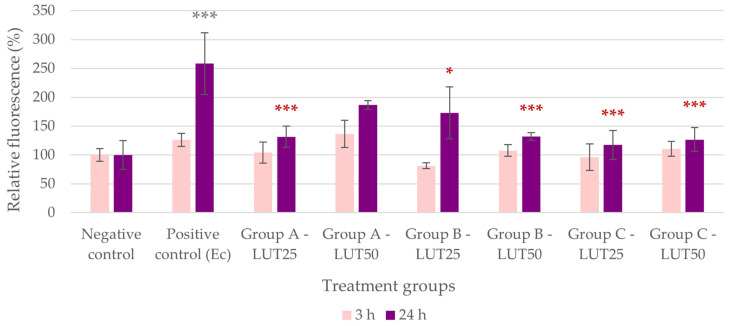
Paracellular permeability of IPEC-J2 cells 3 and 24 h after one-hour treatment with *Escherichia coli* and luteolin (LUT). Negative control: untreated cells (plain medium only); positive control: cells challenged with 10^6^ CFU/mL *E. coli*. Group A—LUT25 or LUT50: cells treated with 25 or 50 μg/mL LUT before challenge with 10^6^ CFU/mL *E. coli*; Group B—LUT25 or LUT50: cells treated with 25 or 50 μg/mL LUT concurrently with challenge with 10^6^ CFU/mL *E. coli*; Group C—LUT25 or LUT50: cells treated with 25 or 50 μg/mL LUT after challenge with 10^6^ CFU/mL *E. coli*. Data are shown as means with standard deviation and expressed as relative fluorescence, considering the mean value of control as 100%. N = 6/group. Significant difference: * *p* < 0.5, *** *p* < 0.001, asterisk in gray: compared to the negative (untreated) control; in red: compared to the positive control (*E. coli* challenge).

Changes in the paracellular permeability of the IPEC-J2 cells were more prominent one day after the treatments compared to the measurement performed only three hours after the treatments. This was probably due to the delayed toxic effect of bacteria. After one day, the paracellular permeability of the cells challenged with bacteria significantly increased, suggesting the barrier-damaging effect of *E. coli* and *S*. Typhimurium infections. When applied together with *E. coli*, LUT could protect the barrier integrity of cells in all cases, except for the 50 μg/mL of LUT used before the bacterial challenge (Group A). The rest of the treatments did not differ in efficacy; all showed a significant barrier-protective effect (Figure 5).

**Figure 6:**
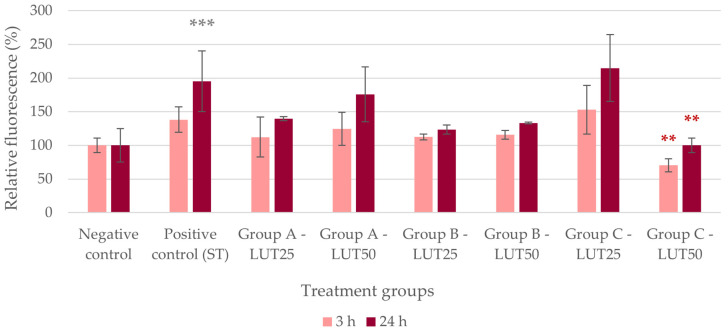
Paracellular permeability of IPEC-J2 cells 3 and 24 h after one-hour treatment with *Salmonella* Typhimurium and luteolin (LUT). Negative control: untreated cells (plain medium only); positive control: cells challenged with 10^6^ CFU/mL *S.* Typhimurium. Group A—LUT25 or LUT50: cells treated with 25 or 50 μg/mL LUT before challenge with 10^6^ CFU/mL *S.* Typhimurium; Group B—LUT25 or LUT50: cells treated with 25 or 50 μg/mL LUT concurrently with challenge with 10^6^ CFU/mL *S.* Typhimurium; Group C—LUT25 or LUT50: cells treated with 25 or 50 μg/mL LUT after challenge with 10^6^ CFU/mL *S.* Typhimurium. Data are shown as means with standard deviation and expressed as relative fluorescence, considering the mean value of control as 100%. N = 6/group. Significant difference: ** *p* < 0.01, *** *p* < 0.001, asterisk in gray: compared to the negative (untreated) control; in red: compared to the positive control (*S.* Typhimurium challenge).

In the case of *S*. Typhimurium infection, most LUT treatments could not alleviate the barrier integrity damage caused by bacteria. Only the higher concentration of LUT (50 μg/mL) showed a significant barrier-protective effect when applied after the challenge with bacteria (Group C). The results are shown in Figure 6.

### 3.4. Adhesion Inhibition

When the IPEC-J2 cells were treated with bacteria without the addition of LUT, the average amount of bacteria that adhered to the cells was 9 × 10^5^ CFU and 2 × 10^5^ CFU for *E. coli* and *S*. Typhimurium, respectively. LUT did not show significant anti-adhesive activity against the tested bacterial strains. When LUT was applied before, concurrently, or after infection with bacteria, a slight reduction was observed in the number of attached bacteria; however, this was not significant in any of the treatment groups.

## 4. Discussion

As bacterial infections can cause significant economic losses to the pig industry, it is of high importance to control the prevalence and severity of these diseases [32]. For instance, *E. coli* and *Salmonella* spp. commonly cause diarrhea in pigs, especially at young ages, leading to loss of productivity [33] and public health concerns, as both bacteria have the potential to cause diseases in humans as well via foodborne infections [34]. In addition, *E. coli* and *Salmonella* spp. isolates—both from human and animal origin—frequently show resistance to antibiotics [34]. The presence of resistant bacteria in microbial communities is a serious concern for animal and human health [35], making AMR one of the main public health threats of the 21st century [36]. Consequently, there is a need to develop alternative strategies for the prevention and treatment of these infections [37] and thus reduce the use of antibiotics in livestock. It has been demonstrated that a reduction in antibiotic use in food-producing animals can decrease the prevalence of AMR in both animals and humans; therefore, this strategy can be effective in combating AMR [11]. The aim of our research was to test the potential beneficial effects of LUT in porcine intestinal infections caused by *E. coli* and *S*. Typhimurium. For this purpose, we used an in vitro model where IPEC-J2 porcine intestinal epithelial cells were challenged with bacteria with or without previous, concurrent, or subsequent additions of LUT and studied the changes in the barrier integrity of the cells, in their ROS and interleukin levels, and in the attachment of bacteria to them. The use of IPEC-J2 cells infected with bacteria has been documented in several studies to model intestinal bacterial infections and to test the protective effects of various substances, including antimicrobial peptides, probiotics, bacteriophages, and flavonoids [9,13,14,26,38,39,40,41,42,43]; however, our research was the first to test the effects of LUT in such a model.

Bacterial infections of the gastrointestinal tract lead to oxidative stress and inflammation in intestinal epithelial cells, thus, the use of antioxidants and anti-inflammatory agents may help to alleviate the disease’s symptoms. The antioxidant and anti-inflammatory effects of LUT have been reported in various studies, however, none of these were conducted in pigs or in cells of porcine origin infected with bacteria. For instance, in an in vivo experiment with chicks, LUT was able to alleviate inflammation, oxidative stress, and intestinal injury caused by infection with avian pathogenic *E. coli*. Similarly to our results, lower IL-6 and IL-8 levels were measured in the LUT-treated groups compared to the ones infected with bacteria without the addition of LUT [18]. In an in vitro study, LUT also showed antioxidant and anti-inflammatory effects against *S*. Typhimurium flagellin-induced oxidative stress and inflammation in a chicken hepatic cell culture. Among other mediators, LUT reduced IL-8 production of cells elevated by the flagellin [44]. LUT could also alleviate inflammation in mouse mammary epithelial cells challenged with *Staphylococcus aureus* (i.e., in a mouse model of *S. aureus* mastitis), in which case, the expression of IL-6 and other mediators were reduced by the flavonoid [45]. Similarly, in the study of Gao et al., LUT showed an anti-inflammatory effect in *S. aureus*-induced endometritis in mice, including a reduction in IL-6 production [46]. Aside from the studies with bacteria, the antioxidant and anti-inflammatory effects of LUT have also been demonstrated in studies with bacterial endotoxins (i.e., lipopolysaccharides, LPS). For instance, LUT could reduce the production of ROS in RAW264.7 murine macrophage cells treated with LPS [47] and the expression of proinflammatory mediators in various in vitro intestine models challenged with LPS [48,49]. Our research group has previously shown the antioxidant effect of LUT in IPEC-J2 cells treated with LPS of *E. coli* and *S*. Typhimurium origin [27]. In the current experiments, LUT alleviated oxidative stress and inflammation by decreasing the ROS, IL-6, and IL-8 levels in IPEC-J2 cells challenged with *E. coli* and *S*. Typhimurium. Our results are in accordance with the literature regarding the antioxidant and anti-inflammatory effects of LUT and demonstrated these properties of LUT in a new in vitro model of porcine intestinal infections.

Aside from causing oxidative stress and inflammation, bacterial infections can disrupt the intestinal barrier integrity, leading to increased permeability and the transport of pathogens to the systemic circulation, thus worsening the disease. The ability of LUT to protect the intestinal barrier, mainly by increasing the expression of tight junction proteins, has been demonstrated in previous studies. This property of the flavonoid was shown in rats, counteracting the effect of a high-fat diet [50,51], and in human colonic adenocarcinoma (Caco-2) cells treated with ethanol [52], polybrominated diphenyl ether [53], and tumor necrosis factor-alpha (TNF-α) and interferon-gamma (IFN-γ) [54]. In our research, LUT could reduce the paracellular permeability of IPEC-J2 cells damaged with *E. coli*, but it seemed to be less effective against the barrier disruption caused by *S*. Typhimurium. To better understand the observed difference between the effect of LUT against *E. coli* and *S*. Typhimurium, there is a need to further study the underlying mechanisms of the flavonoid’s barrier-protective activity in intestinal inflammations.

The attachment of bacteria to intestinal epithelial cells is the first step toward intestinal infections. Consequently, inhibiting the adhesion of bacteria to the cells could prevent the development of diarrhea. There are some reports about the potential of LUT to inhibit the adhesion of microorganisms, however, it has not yet been widely documented. For instance, Fu et al. observed that LUT could reduce the attachment and biofilm formation of *Candida albicans* and *Enterococcus faecalis* to glass surfaces [55]. Shen et al. reported that the flavonoid inhibited adhesion and invasion of uropathogenic *E. coli* to human bladder epithelial cells [56]. In addition, Šikić Pogačar et al. observed that various plant extracts that contained luteolin 7-O-glucoside and other phytochemicals could inhibit the adhesion of *Campylobacter jejuni* to abiotic surfaces and porcine small intestinal epithelial cells [57]. In contrast to these findings, we could not detect the anti-adhesive activity of LUT against *E. coli* and *S*. Typhimurium in IPEC-J2 cells. This discrepancy could be due to the different bacterial strains and host cells being tested in our research compared to previous experiments. Nevertheless, there is a need to further study the anti-adhesive effect of LUT, and the underlying mechanisms that could shed light on the differences in its activity between different experimental settings.

It has been previously demonstrated that LUT has an antibacterial effect against *E. coli* and *S*. Typhimurium. In previous experiments, the minimum inhibitory concentration (MIC) values of LUT ranged between 200 and 2500 μg/mL against *E. coli* and between 256 and 1250 μg/mL against *S*. Typhimurium isolates [27,58,59,60]. In our research, the observed beneficial effects of LUT (antioxidant, anti-inflammatory, and barrier-protective effects) occurred at remarkably lower concentrations (25–50 μg/mL) than the direct antibacterial effect of LUT in previous experiments. Consequently, the antibacterial activity of LUT is not likely to be in the background of the observed beneficial effects.

When comparing the efficacy of LUT applied in different concentrations and at different times (before, concurrently, or after challenge with bacteria), we could not observe a clear dose–effect relationship or any of the treatment types being clearly superior to the others. Further studies are needed to characterize the mechanism of action of LUT against bacterial infections and to identify the optimal concentration and treatment type. In addition, there is a need for in vivo experiments to confirm the results of the in vitro studies for the future use of LUT in intestinal diseases of pigs.

## 5. Conclusions

Intestinal infections caused by *E. coli* and *S*. Typhimurium lead to loss of productivity and increased mortality in pigs, thus posing an economic burden on the swine industry. These infections are generally treated with antibiotics; however, due to the development and spread of antimicrobial resistance in these pathogens, alternative prevention and treatment strategies need to be considered. In this research, we demonstrated that LUT can counteract the deleterious effects of bacteria in an in vitro model of porcine intestinal infections. LUT could alleviate oxidative stress, inflammation, and barrier integrity disruption caused by *E. coli* and *S*. Typhimurium infections in porcine intestinal epithelial cells. Based on these findings, LUT is a potential candidate to be used in *E. coli* and *S*. Typhimurium infections in pigs to counteract the damaging effect of bacteria in the intestines. The observed effects of LUT can be beneficial if the flavonoid will be used against these infections in the future, ultimately leading to a reduced need for antibiotics in pig production. However, further in vitro and in vivo studies are needed to better understand its mechanism of action and efficacy.

## Figures and Tables

**Table 1:** Treatment scheme of the experiments.

	Step 1	Step 2		Step 3
**Positive control**	Addition of bacteria		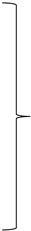	Measurements Reactive oxygen species level Interleukin-6 and interleukin-8 levels Paracellular permeability Bacterial adhesion
**Group A** LUT treatment ***before*** challenge with bacteria	Addition of LUT	Addition of bacteria		
**Group B** LUT treatment ***concurrently*** with challenge with bacteria	Addition of LUT and bacteria			
**Group C** LUT treatment ***after*** challenge with bacteria	Addition of bacteria	Addition of LUT		
**Negative control**	Addition of plain medium			

LUT: luteolin 25 or 50 µg/mL. Bacteria: *Escherichia coli* or *Salmonella* Typhimurium, 10^6^ CFU/mL.

## Data Availability

Data of this research are available at the authors upon request.

## References

[1] Dubreuil J.D., Isaacson R.E., Schifferli D.M. (2016). Animal Enterotoxigenic *Escherichia coli*. EcoSal Plus.

[2] Kim K., Song M., Liu Y., Ji P. (2022). Enterotoxigenic *Escherichia coli* infection of weaned pigs: Intestinal challenges and nutritional intervention to enhance disease resistance. Front. Immunol..

[3] Kim H.B., Isaacson R.E. (2017). *Salmonella* in Swine: Microbiota Interactions. Annu. Rev. Anim. Biosci..

[4] Gresse R., Chaucheyras-Durand F., Fleury M.A., Van de Wiele T., Forano E., Blanquet-Diot S. (2017). Gut Microbiota Dysbiosis in Postweaning Piglets: Understanding the Keys to Health. Trends Microbiol..

[5] Rhouma M., Fairbrother J.M., Beaudry F., Letellier A. (2017). Post weaning diarrhea in pigs: Risk factors and non-colistin-based control strategies. Acta Vet. Scand..

[6] Su W., Gong T., Jiang Z., Lu Z., Wang Y. (2022). The Role of Probiotics in Alleviating Postweaning Diarrhea in Piglets from the Perspective of Intestinal Barriers. Front. Cell Infect. Microbiol..

[7] Tran T.H.T., Everaert N., Bindelle J. (2018). Review on the effects of potential prebiotics on controlling intestinal enteropathogens *Salmonella* and *Escherichia coli* in pig production. J. Anim. Physiol. Anim. Nutr..

[8] Wijtten P.J., van der Meulen J., Verstegen M.W. (2011). Intestinal barrier function and absorption in pigs after weaning: A review. Br. J. Nutr..

[9] Li Q., Li L., Chen Y., Yu C., Azevedo P., Gong J., Yang C. (2022). *Bacillus licheniformis* PF9 improves barrier function and alleviates inflammatory responses against enterotoxigenic *Escherichia coli* F4 infection in the porcine intestinal epithelial cells. J. Anim. Sci. Biotechnol..

[10] Ter Kuile B.H., Kraupner N., Brul S. (2016). The risk of low concentrations of antibiotics in agriculture for resistance in human health care. FEMS Microbiol. Lett..

[11] Tang K.L., Caffrey N.P., Nóbrega D.B., Cork S.C., Ronksley P.E., Barkema H.W., Polachek A.J., Ganshorn H., Sharma N., Kellner J.D. (2017). Restricting the use of antibiotics in food-producing animals and its associations with antibiotic resistance in food-producing animals and human beings: A systematic review and meta-analysis. Lancet Planet. Health.

[12] Emes D., Naylor N., Waage J., Knight G. (2022). Quantifying the Relationship between Antibiotic Use in Food-Producing Animals and Antibiotic Resistance in Humans. Antibiotics.

[13] Fu Q., Lin Q., Chen D., Yu B., Luo Y., Zheng P., Mao X., Huang Z., Yu J., Luo J. (2022). β-defensin 118 attenuates inflammation and injury of intestinal epithelial cells upon enterotoxigenic *Escherichia coli* challenge. BMC Vet. Res..

[14] Kim N., Gu M.J., Kye Y.C., Ju Y.J., Hong R., Ju D.B., Pyung Y.J., Han S.H., Park B.C., Yun C.H. (2022). Bacteriophage EK99P-1 alleviates enterotoxigenic *Escherichia coli* K99-induced barrier dysfunction and inflammation. Sci. Rep..

[15] Ponce de León-Rodríguez M.D.C., Guyot J.P., Laurent-Babot C. (2019). Intestinal in vitro cell culture models and their potential to study the effect of food components on intestinal inflammation. Crit. Rev. Food Sci. Nutr..

[16] Vergauwen H., Verhoeckx K., Cotter P., López-Expósito I., Kleiveland C., Lea T., Mackie A., Requena T., Swiatecka D., Wichers H. (2015). The IPEC-J2 Cell Line. The Impact of Food Bioactives on Health: In Vitro and Ex Vivo Models.

[17] Berschneider H.M. (1989). Development of normal cultured small intestinal epithelial cell lines which transport Na and Cl. Gastroenterology.

[18] Cao Z., Xing C., Cheng X., Luo J., Hu R., Cao H., Guo X., Yang F., Zhuang Y., Hu G. (2022). Luteolin Attenuates APEC-Induced Oxidative Stress and Inflammation via Inhibiting the HMGB1/TLR4/NF-κB Signal Axis in the Ileum of Chicks. Animals.

[19] Caporali S., De Stefano A., Calabrese C., Giovannelli A., Pieri M., Savini I., Tesauro M., Bernardini S., Minieri M., Terrinoni A. (2022). Anti-Inflammatory and Active Biological Properties of the Plant-Derived Bioactive Compounds Luteolin and Luteolin 7-Glucoside. Nutrients.

[20] Chagas M.D.S.S., Behrens M.D., Moragas-Tellis C.J., Penedo G.X.M., Silva A.R., Gonçalves-de-Albuquerque C.F. (2022). Flavonols and Flavones as Potential Anti-Inflammatory, Antioxidant, and Antibacterial Compounds. Oxid. Med. Cell Longev..

[21] Muruganathan N., Dhanapal A.R., Baskar V., Muthuramalingam P., Selvaraj D., Aara H., Shiek Abdullah M.Z., Sivanesan I. (2022). Recent Updates on Source, Biosynthesis, and Therapeutic Potential of Natural Flavonoid Luteolin: A Review. Metabolites.

[22] Rakha A., Umar N., Rabail R., Butt M.S., Kieliszek M., Hassoun A., Aadil R.M. (2022). Anti-inflammatory and anti-allergic potential of dietary flavonoids: A review. Biomed. Pharmacother..

[23] Ju W., Wang X., Shi H., Chen W., Belinsky S.A., Lin Y. (2007). A critical role of luteolin-induced reactive oxygen species in blockage of tumor necrosis factor-activated nuclear factor-kappaB pathway and sensitization of apoptosis in lung cancer cells. Mol. Pharmacol..

[24] Wang Q., Wang H., Jia Y., Pan H., Ding H. (2017). Luteolin induces apoptosis by ROS/ER stress and mitochondrial dysfunction in gliomablastoma. Cancer Chemother. Pharmacol..

[25] Matsuo M., Sasaki N., Saga K., Kaneko T. (2005). Cytotoxicity of flavonoids toward cultured normal human cells. Biol. Pharm. Bull..

[26] Kovács D., Palkovicsné Pézsa N., Jerzsele Á., Süth M., Farkas O. (2022). Protective Effects of Grape Seed Oligomeric Proanthocyanidins in IPEC-J2-*Escherichia coli*/*Salmonella* Typhimurium Co-Culture. Antibiotics.

[27] Kovács D., Karancsi Z., Farkas O., Jerzsele Á. (2020). Antioxidant activity of flavonoids in LPS-treated IPEC-J2 porcine intestinal epithelial cells and their antibacterial effect against bacteria of swine origin. Antioxidants.

[28] Wang H., Joseph J.A. (1999). Quantifying cellular oxidative stress by dichlorofluorescein assay using microplate reader. Free Radic. Biol. Med..

[29] Loss H., Aschenbach J.R., Tedin K., Ebner F., Lodemann U. (2018). The inflammatory response to enterotoxigenic *E. coli* and probiotic *E. faecium* in a coculture model of porcine intestinal epithelial and dendritic cells. Mediat. Inflamm..

[30] Loss H., Aschenbach J.R., Ebner F., Tedin K., Lodemann U. (2020). Inflammatory Responses of Porcine MoDC and Intestinal Epithelial Cells in a Direct-Contact Co-culture System Following a Bacterial Challenge. Inflammation.

[31] Karancsi Z., Móritz A.V., Lewin N., Veres A.M., Jerzsele Á., Farkas O. (2020). Beneficial Effect of a Fermented Wheat Germ Extract in Intestinal Epithelial Cells in case of Lipopolysaccharide-Evoked Inflammation. Oxid. Med. Cell Longev..

[32] Lückstädt C., Theobald P. Control of *E. coli* and *Salmonella* in growing-finishing pigs through the use of potassium diformate (KDF)—European case studies. Proceedings of the Ninth International Conference on the Epidemiology and Control of Biological, Chemical and Physical Hazards in Pigs and Pork.

[33] Edfors-Lilja I., Wallgren P., Axford R.F.E., Bishop S.C., Nicholas F.W., Owen J.B. (2000). Escherichia coli and Salmonella Diarrhoea in Pigs, Breeding for Disease Resistance in Farm Animals.

[34] Souto M.S., Coura F.M., Dorneles E.M., Stynen A.P., Alves T.M., Santana J.A., Pauletti R.B., Guedes R.M., Viott A.M., Heinemann M.B. (2017). Antimicrobial susceptibility and phylotyping profile of pathogenic *Escherichia coli* and *Salmonella enterica* isolates from calves and pigs in Minas Gerais, Brazil. Trop. Anim. Health Prod..

[35] European Food Safety Authority (EFSA), European Centre for Disease Prevention and Control (ECDC) (2021). The European Union Summary Report on Antimicrobial Resistance in zoonotic and indicator bacteria from humans, animals and food in 2018/2019. EFSA J..

[36] Antimicrobial Resistance Collaborators (2022). Global burden of bacterial antimicrobial resistance in 2019: A systematic analysis. Lancet.

[37] Luppi A. (2017). Swine enteric colibacillosis: Diagnosis, therapy and antimicrobial resistance. Porc. Health Manag..

[38] Gao J., Cao S., Xiao H., Hu S., Yao K., Huang K., Jiang Z., Wang L. (2022). *Lactobacillus reuteri* 1 Enhances Intestinal Epithelial Barrier Function and Alleviates the Inflammatory Response Induced by Enterotoxigenic *Escherichia coli* K88 via Suppressing the MLCK Signaling Pathway in IPEC-J2 Cells. Front. Immunol..

[39] Kiššová Z., Tkáčiková L., Mudroňová D., Bhide M.R. (2022). Immunomodulatory Effect of *Lactobacillus reuteri* (*Limosilactobacillus reuteri*) and Its Exopolysaccharides Investigated on Epithelial Cell Line IPEC-J2 Challenged with *Salmonella* Typhimurium. Life.

[40] Zhang K., Lian S., Shen X., Zhao X., Zhao W., Li C. (2022). Recombinant porcine beta defensin 2 alleviates inflammatory responses induced by *Escherichia coli* in IPEC-J2 cells. Int. J. Biol. Macromol..

[41] Palkovicsné Pézsa N., Kovács D., Somogyi F., Karancsi Z., Móritz A.V., Jerzsele Á., Rácz B., Farkas O. (2023). Effects of *Lactobacillus rhamnosus* DSM7133 on Intestinal Porcine Epithelial Cells. Animals.

[42] Palkovicsné Pézsa N., Kovács D., Rácz B., Farkas O. (2022). Effects of *Bacillus licheniformis* and *Bacillus subtilis* on Gut Barrier Function, Proinflammatory Response, ROS Production and Pathogen Inhibition Properties in IPEC-J2—*Escherichia coli*/*Salmonella* Typhimurium Co-Culture. Microorganisms.

[43] Palkovicsné Pézsa N., Kovács D., Gálfi P., Rácz B., Farkas O. (2022). Effect of *Enterococcus faecium* NCIMB 10415 on Gut Barrier Function, Internal Redox State, Proinflammatory Response and Pathogen Inhibition Properties in Porcine Intestinal Epithelial Cells. Nutrients.

[44] Tráj P., Sebők C., Mackei M., Kemény Á., Farkas O., Kákonyi Á., Kovács L., Neogrády Z., Jerzsele Á., Mátis G. (2023). Luteolin: A Phytochemical to Mitigate *S*. Typhimurium Flagellin-Induced Inflammation in a Chicken In Vitro Hepatic Model. Animals.

[45] Guo Y.F., Xu N.N., Sun W., Zhao Y., Li C.Y., Guo M.Y. (2017). Luteolin reduces inflammation in *Staphylococcus aureus*-induced mastitis by inhibiting NF-kB activation and MMPs expression. Oncotarget.

[46] Gao S., Gao Y., Cai L., Qin R. (2024). Luteolin attenuates *Staphylococcus aureus*-induced endometritis through inhibiting ferroptosis and inflammation via activating the Nrf2/GPX4 signaling pathway. Microbiol. Spectr..

[47] Zhang B.C., Li Z., Xu W., Xiang C.H., Ma Y.F. (2018). Luteolin alleviates NLRP3 inflammasome activation and directs macrophage polarization in lipopolysaccharide-stimulated RAW264.7 cells. Am. J. Transl. Res..

[48] Nishitani Y., Yamamoto K., Yoshida M., Azuma T., Kanazawa K., Hashimoto T., Mizuno M. (2013). Intestinal anti-inflammatory activity of luteolin: Role of the aglycone in NF-κB inactivation in macrophages co-cultured with intestinal epithelial cells. Biofactors.

[49] Kim J.S., Jobin C. (2005). The flavonoid luteolin prevents lipopolysaccharide-induced NF-kappaB signalling and gene expression by blocking IkappaB kinase activity in intestinal epithelial cells and bone-marrow derived dendritic cells. Immunology.

[50] Liu X., Sun R., Li Z., Xiao R., Lv P., Sun X., Olson M.A., Gong Y. (2021). Luteolin alleviates non-alcoholic fatty liver disease in rats via restoration of intestinal mucosal barrier damage and microbiota imbalance involving in gut-liver axis. Arch. Biochem. Biophys..

[51] Sun W.L., Yang J.W., Dou H.Y., Li G.Q., Li X.Y., Shen L., Ji H.F. (2021). Anti-inflammatory effect of luteolin is related to the changes in the gut microbiota and contributes to preventing the progression from simple steatosis to nonalcoholic steatohepatitis. Bioorg. Chem..

[52] Yuan J., Che S., Zhang L., Ruan Z. (2021). Reparative Effects of Ethanol-Induced Intestinal Barrier Injury by Flavonoid Luteolin via MAPK/NF-κB/MLCK and Nrf2 Signaling Pathway. J. Agric. Food Chem..

[53] Yuan J., Che S., Ruan Z., Song L., Tang R., Zhang L. (2021). Regulatory effects of flavonoids luteolin on BDE-209-induced intestinal epithelial barrier damage in Caco-2 cell monolayer model. Food Chem. Toxicol..

[54] Li B.L., Zhao D.Y., Du P.L., Wang X.T., Yang Q., Cai Y.R. (2021). Luteolin alleviates ulcerative colitis through SHP-1/STAT3 pathway. Inflamm. Res..

[55] Fu Y., Wang W., Zeng Q., Wang T., Qian W. (2021). Antibiofilm Efficacy of Luteolin Against Single and Dual Species of *Candida albicans* and *Enterococcus faecalis*. Front. Microbiol..

[56] Shen X.F., Ren L.B., Teng Y., Zheng S., Yang X.L., Guo X.J., Wang X.Y., Sha K.H., Li N., Xu G.Y. (2014). Luteolin decreases the attachment, invasion and cytotoxicity of UPEC in bladder epithelial cells and inhibits UPEC biofilm formation. Food Chem. Toxicol..

[57] Šikić Pogačar M., Klančnik A., Bucar F., Langerholc T., Smole Možina S. (2016). Anti-adhesion activity of thyme (*Thymus vulgaris* L.) extract, thyme post-distillation waste, and olive (*Olea europea* L.) leaf extract against *Campylobacter jejuni* on polystyrene and intestine epithelial cells. J. Sci. Food Agric..

[58] Sanhueza L., Melo R., Montero R., Maisey K., Mendoza L., Wilkens M. (2017). Synergistic interactions between phenolic compounds identified in grape pomace extract with antibiotics of different classes against *Staphylococcus aureus* and *Escherichia coli*. PLoS ONE.

[59] Huang C.C., Gao X., Sun T.T., Yu L., Guo Y., Hong W., Zhang D., Liu M. (2017). The antimicrobial activity of luteolin against four bacteria in vitro. Chin. J. Vet. Sci..

[60] Adamczak A., Ożarowski M., Karpiński T.M. (2019). Antibacterial Activity of Some Flavonoids and Organic Acids Widely Distributed in Plants. J. Clin. Med..

